# 

**Published:** 1910-09

**Authors:** 


SEPTEMBER, 1910.

*Vol. XXVIII.* *No.* 109.

LIBRARY
SURGEON GENERAL'S OFFICE
OCT 7 - 1910

### THE BRISTOL MEDICO-CHIRURGICAL JOURNAL

*PUBLISHED UNDER THE AUSPICES OF*

*THE BRISTOL MEDICO-CHIRURGICAL SOCIETY.*

*Editor:* R. SHINGLETON SMITH, M.D., B.Sc.

WITH WHOM ARE ASSOCIATED

J. MICHELL CLARKE, M.A., M.D.,

J. LACY FIRTH, M.S., M.D., JAMES SWAIN, M.S., M.D.

P. WATSON WILLIAMS, M.D., *Assistant Editor.*

*Editorial Secretary:* J. A. NIXON, B.A., M.B.

BRISTOL: J. W. ARROWSMITH.
London: J. & A. Churchill, 7 Great Marlborough Street.

*Price One Shilling and Sixpence.*

